# Organ homologies in orchid flowers re-interpreted using the Musk Orchid as a model

**DOI:** 10.7717/peerj.26

**Published:** 2013-02-12

**Authors:** Paula J. Rudall, Craig D. Perl, Richard M. Bateman

**Affiliations:** Royal Botanic Gardens Kew, Richmond, Surrey, United Kingdom

**Keywords:** Congenital fusion, B-class genes, Floral ontogeny, Gynostemium, *Herminium*, *KNOX*-gene expression, Labellum, Orchid, Partial homology, Synorganization

## Abstract

**Background and Aims**. The presence of novel structures in orchid flowers, including auricles, rostellum and bursicles on the gynostemium and a lobed labellum, has prompted long-standing homology disputes, fuelled by conflicting evidence from a wide range of sources. Re-assessment of this debate using an improved model is timely, following recent phylogenetic insights and on the cusp of a revolution in developmental genetics.

**Methods**. We use new data from floral development and anatomy in the small-flowered terrestrial orchid *Herminium monorchis* as a model to explore organ homologies in orchid flowers within the context of a review of recent literature on developmental genetics.

**Key Results**. The apex of the median carpel of *Herminium* is trilobed, and the bursicles develop from its lateral lobes, relatively late in flower ontogeny. The bursicles enclose the viscidia, which adhere to the tapetal remnants to form a caudicle linking the viscidium with the pollinium. The auricles are initiated earlier than the bursicles, but they also remain unvascularized. The deeply trilobed labellum possesses three vascular traces, in contrast with the lateral petals, each of which contains a single vascular trace. The two lateral labellum traces diverge from the traces supplying the two adjacent lateral sepals. Data from flower ontogeny and anatomy conflict with respect to organ homologies.

**Conclusions**. Much progress has recently been made in understanding the exceptional differentiation shown by orchids among perianth segments, focusing on multiple copies of the *DEF/AP3* subclass of B-class MADS-box genes. In contrast, untangling homologies of profound congenital union of multiple floral organs forming the orchid gynostemium is hampered by their profound congenital union, which we ascribe to overlap in gene expression between organs. Thus, the functional morphology of the orchid flower could ultimately reflect extreme synorganization and associated genetic integration. Analogizing the deeply lobed orchid labellum with a compound leaf, we speculate that *KNOX* genes could be implicated not only in their demonstrated role in spur development but also in the development of both the characteristic lobed morphology of the orchid labellum and the lobing of the median carpel that differentiates the bursicles and rostellum.

## Introduction

### Unique architecture of the orchid flower

**Figure 1:**
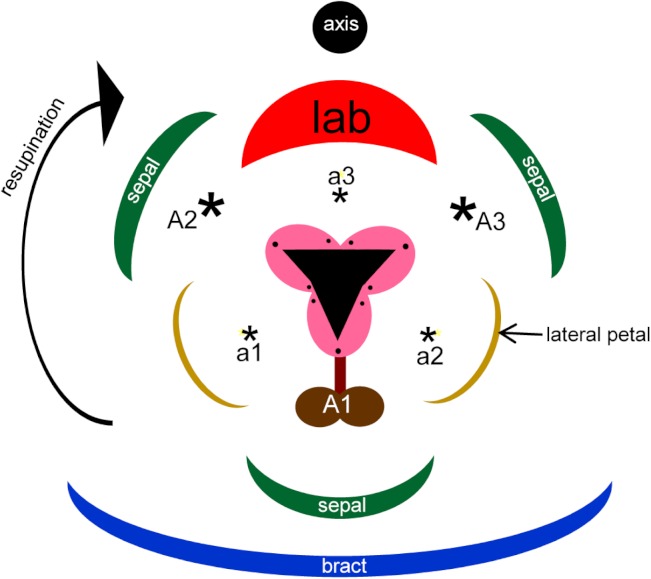
Floral diagram of *Herminium monorchis*, typical of orchidoid orchids. Asterisks indicate expected locations of missing stamens, based on comparison with a typical trimerous–pentacyclic monocot flower (e.g. Rudall & Bateman, 2002). Abbreviations: a = inner-whorl stamens, A = outer-whorl stamens, lab = labellum.

Flowers of orchids possess a unique combination of features that together distinguish them from all other monocots, and indeed from all other flowering plants (reviewed in detail by Rudall & Bateman, 2002). They are epigynous and show strong bilateral symmetry (monosymmetry) of both perianth and fertile organs, typically associated with 180° torsion (resupination) and with sterilization or complete suppression of the majority of the six ancestral stamens. The remaining fertile organs are congenitally united into a gynostemium, a complex structure that has long demanded greater attention from evolutionary-developmental geneticists (Johansen & Frederiksen, 2002; Rudall & Bateman, 2002). Orchid flowers also possess some prominent and apparently novel appendages on the gynostemium, such as auricles and bursicles. Auricles are a pair of sterile outgrowths that are lateral to, and separated by, the gynostemium. They are typically rich in raphides – bundles of acicular crystals of calcium oxalate enclosed within a single cell. A bursicle is a thin sac-like membrane that covers the viscidial discs responsible for adhering the pollinaria to pollinating insects. In the two most species-rich and evolutionarily derived orchid subfamilies, Orchidoideae and Epidendroideae, the fertile organs consist of a single stamen (the median stamen of the outer whorl: A1) and three carpels (Fig. 1). All other ancestral stamens – two lateral outer-whorl stamens, A2 and A3, and all three inner-whorl stamens, a1, a2 and a3 – are either totally suppressed or putatively (and controversially) expressed as sterile appendages.

Furthermore, the orchid perianth is differentiated into two closely-spaced but nonetheless distinct whorls of three tepals each. Although both of the tepal whorls are often coloured, in orchids they are commonly termed sepals and petals respectively. A differentiated perianth is a common condition in eudicots but relatively infrequent in monocots, where it has originated several times (e.g. Remizowa et al., 2012). Finally, the median petal in orchids, termed the labellum, differs in size from the two lateral petals. The labellum is usually larger and more elaborate in both shape and markings; it is frequently deeply lobed and in some species, especially in subfamily Orchidoideae, it is invaginated toward the proximal end to form a spur (e.g. Bell et al., 2009).

The presence of putatively novel structures, coupled with the profound fusion of some reproductive organs and apparent loss of others, makes interpretation of organ homologies especially problematic in orchid flowers. Indeed, the question of whether the orchid labellum is a compound structure formed by total integration of more than one organ – here termed the compound labellum hypothesis – remains one of the most enduring homology debates concerning any plant organ, despite its unequivocal dismissal by some authors (e.g. Yam, Arditti & Cameron, 2009). Since the mid-nineteenth century there has been considerable discussion of the various processes potentially leading to organ reduction and fusion in orchid flowers (cf. Brown, 1833; Darwin, 1862; Carlquist, 1969; Wilson, 1982; Rudall & Bateman, 2002). Competing hypotheses have been based on several lines of evidence that often appear contradictory, including natural teratology (Brown, 1833; Vermeulen, 1953; Vermeulen, 1966; Bateman & Rudall, 2006), ontogeny (Kurzweil, 1987a; Kurzweil, 1987b; Kurzweil, 1988; Kurzweil, 1998; Luo & Chen, 2000; Kocyan & Endress, 2001), vasculature (Swamy, 1948; Nelson, 1965; Nelson, 1967) and relative organ topology (e.g. Ronse Decraene & Smets, 2001). Similar homology issues arise in other monocot groups such as *Corsia* and gingers (Rudall & Bateman, 2002; Rudall & Bateman, 2004). Following recent developments in both phylogenetics and developmental genetics of orchids and related monocots, reassessment of this debate is timely.

### *Herminium* as a new model system

**Figure 2:**
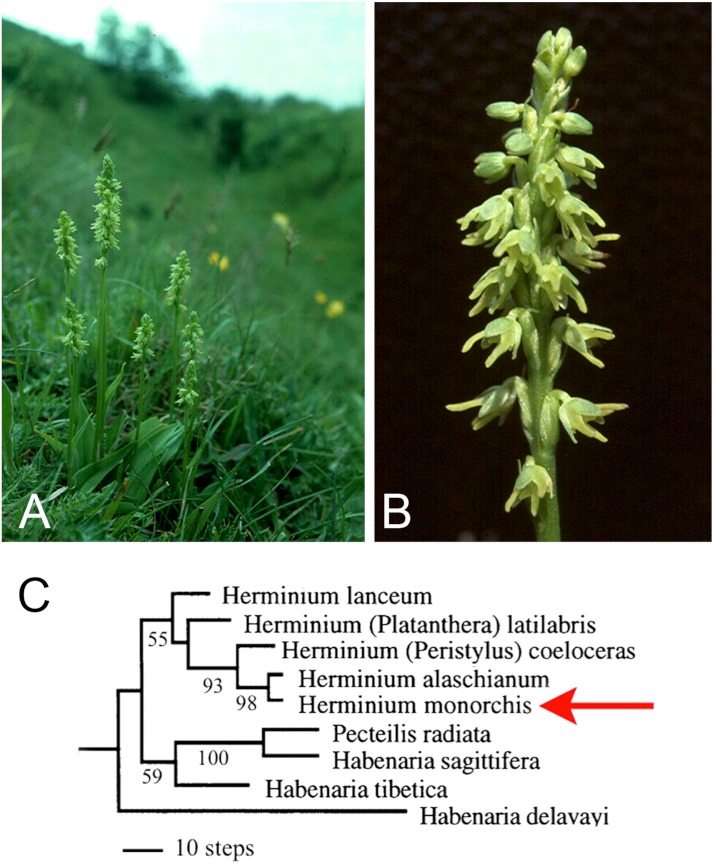
(A) Entire plants and (B) magnified inflorescence of *Herminium monorchis* R.Br. from chalk grassland at Totternhoe Knolls, Bedfordshire. (C) Portion of molecular phylogeny based on the nuclear ribosomal Internal Transcribed Spacer (ITS) region (Bateman et al., 2003), showing relationships of *Herminium* and related habenariids. Numbers represent percentage bootstrap support.

Here, new data from one of the least showy European terrestrial orchids, *Herminium monorchis* R.Br. (Musk Orchid: Fig. 2), provide the focus of our reappraisal of the various lines of evidence that have been used to interpret the homologies of orchid flowers. We then evaluate the potential for future resolution of these perennial questions, particularly using developmental genetics. Despite its phylogenetic placement in a relatively derived orchid subfamily, Orchidoideae, *H. monorchis* represents a useful model for discussing homologies, partly because – unusually for Eurasian terrestrial orchids – it reliably exhibits a broad range of developmental stages on a single above-ground inflorescence. By contrast, in most other orchidoids, all of the floral organs are well-developed before the inflorescence emerges above the soil surface. Thus, early stages of flower development are relatively accessible in *H. monorchis*. Furthermore, despite its small size (flowers rarely exceed 4 mm in diameter: Fig. 2) and relatively undifferentiated perianth, the flower of *H. monorchis* possesses prominent bursicles and auricles, which provide a suitable basis for detailed investigation. We use our observations on the floral ontogeny of *H. monorchis* to review the evidence for contrasting interpretations of the broader homologies of floral organs in orchids *per se*. In particular, we explore three primary hypotheses that seek to explain the “missing” stamens in orchid flowers: (1) the compound labellum hypothesis, in which the missing outer-whorl lateral stamens (A2, A3) are putatively integrated into the labellum; (2) the bursicle hypothesis, where either A2 and A3 or the missing inner-whorl lateral stamens (a1, a2) are expressed as bursicles; and (3) the auricle hypothesis, where a1 and a2 are expressed as auricles.

### Functional morphology and evolutionary origin of the *Herminium* flower

The genus *Herminium* L. belongs in tribe Orchideae, subtribe Habenariinae of the monandrous subfamily Orchidoideae. The 20–30 species are morphologically cohesive, though they encompass a surprising diversity of chromosome numbers (Luo, 2004). Nuclear ribosomal ITS sequencing (Bateman et al., 2003) indicated that the genus is monophyletic and nested among species belonging to several poorly delimited genera of habenariids that collectively are paraphyletic relative to the monophyletic subtribe Orchidinae s.s. (Fig. 2C). *Herminium* is wholly Asiatic and temperate/alpine, with the partial exception of the unusually widespread Eurasiatic species *H. monorchis* (Pridgeon et al., 2001).

The pollination biology and functional morphology of the flower of *H. monorchis* were described by Darwin (1877), though he failed to note the secretion of modest quantities of nectar from two narrow green swellings at the sides of the exceptionally short, saccate spur (Hagerup, 1952; Nilsson, 1979; Pridgeon et al., 2001; Claessens & Kleynen, 2011). Combining the nectar with the strong honey-like scent allows the flowers to attract a wide range of pollinators, despite being small and green. Nilsson (1979) recorded several species of insect visiting a single Swedish population of musk orchids, spanning a remarkable range of small flies, beetles and solitary wasps. Most visitors removed at least some pollinaria. The campanulate flower shape and near-radially symmetrical perianth mean that the labellum does not offer an adequate landing stage, obliging insects to approach the nectar source (and thus the gynostemium) obliquely. Consequently, most visitors remove only one of the two pollinaria, which typically becomes attached to one of the insect’s legs. Although efficient (an average capsule-set of *ca* 80% was reported by Claessens & Kleynen, 2011), this rather ponderous pollination mechanism tends to result in fertilization of other flowers on the same inflorescence that provided the pollinarium – genetically, this pattern, termed geitonogamy, equates with self-pollination. When considered alongside the rapid annual population expansion achieved vegetatively through multiple stolons, these observations arguably undermine Darwin’s (1877) use of *H. monorchis* as a supposedly classic exemplar of cross-pollination engendering high (genetic) diversity. On the other hand, Nilsson (1979) comprehensively refuted Hagerup’s (1952) assertion that the species is facultatively autogamous.

Some earlier authors mistakenly concluded that *Herminium* is “primitive” within tribe Orchideae or represents a “connecting link” between other, better-known taxa (e.g. Hagerup, 1952) – an inference partly based on features such as the small, green flowers (Fig. 2B), the presence of stomata on the adaxial surface of the sepals, the relatively poor differentiation of the labellum (resulting in a near-radially symmetrical perianth) and the exceptionally short spur. However, this hypothesis is refuted by the position of *Herminium* in molecular phylogenies (e.g. Fig. 2C), where it reliably nests among species of *Habenaria s.l*. Comparison with these *Habenaria* species suggests that the floral morphology of *Herminium* reflects a combination of dwarfism and paedomorphic heterochrony – retention of juvenile features of the ancestor in mature individuals of the descendant. Differentiation of the labellum and associated spur barely exceeds that of the lateral petals, elongation of the gynostemium is minimal, and the sepal stomata resemble those present on the subtending bracts. Such simplification and miniaturization of flowers is a recurrent evolutionary theme among alpine orchids of temperate Eurasia; for example, a broadly similar relationship apparently exists between the apparently paraphyletic *Gymnadenia s.s.* and unquestionably monophyletic *“Nigritella”* (e.g. Bateman & DiMichele, 2002).

## Materials and Methods

Flowers and buds of *H. monorchis* were sampled from emergent inflorescences in the seed-based collections of private orchid growers. Reluctance to destroy individual plants dissuaded us from excavating early-stage inflorescences enclosed within the tubers in spring. Material preserved in 70% ethanol was processed for both scanning electron microscopy (SEM) and light microscopy (LM). For SEM, buds were removed from the inflorescence and dehydrated through an ethanol series to 100% ethanol. Samples were dried in a Tousimis Autosamdri 815B critical-point dryer (CPD) using carbon dioxide as the carrier gas. Flowers were mounted onto stubs using double-sided adhesive discs and dissected under a Wild Heerbrugg M7A microscope. Dissected samples were coated in platinum using an Emitech K550 sputter coater and imaged using a Hitachi S-4700 II cold-field emission scanning electron microscope (SEM). Sections were prepared for light microscopy using Technovit 7100 resin. Sections were cut at 6 µm thickness using a Leica RM2155 microtome, mounted in water, dried and then stained with 0.5% (w/v) solution of toluidine blue. Coverslips were mounted using DPX mountant. Images were captured using a Zeiss Axiocam HRc camera attached to a Leica DMLB microscope.

## Results

### Early development (Figs. 3, 4)

**Figure 3:**
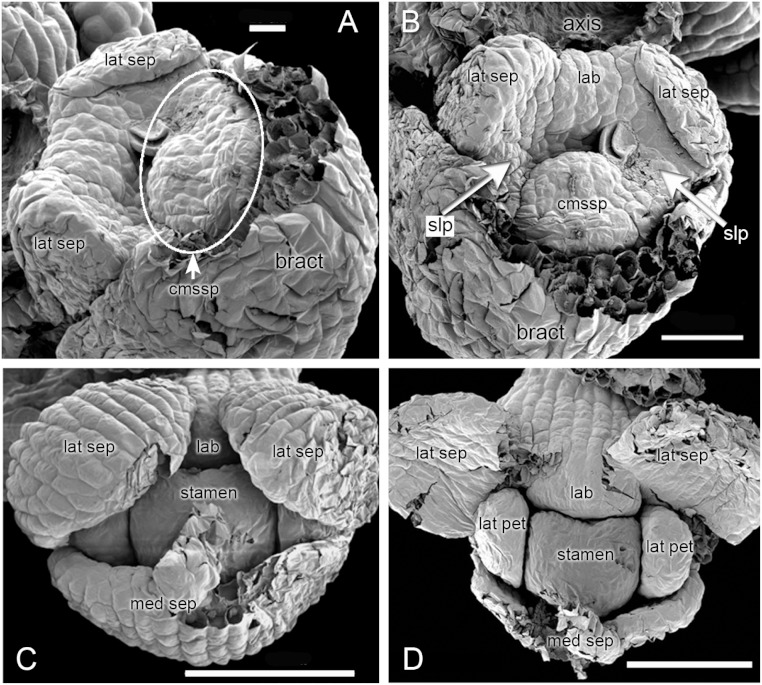
*Herminium monorchis*, SEM dissected flower buds at early stages. (A, B) Two contrasting views of the same bud. (C, D) Two phases in dissection of another bud at a slightly later stage. Abbreviations: cmssp = compound stamen–median sepal primordium, lab = labellum primordium, lat pet = lateral petal primordium, lat sep = lateral sepal primordium, med sep = median sepal primordium, slp = site of lateral petals. Scales: A = 20 µm, B = 50 µm, C, D = 100 µm.

**Figure 4:**
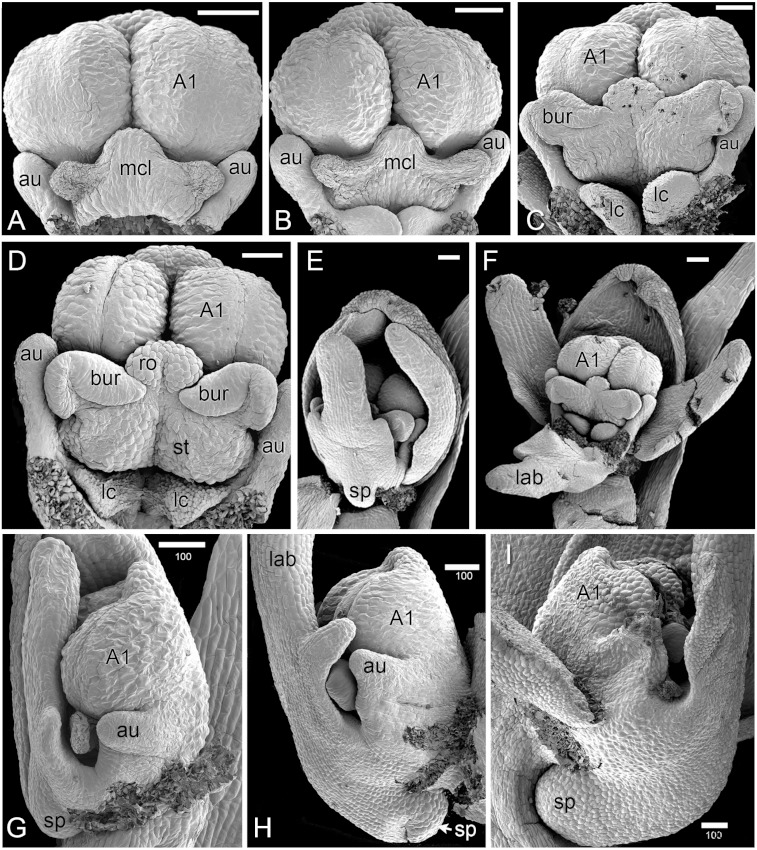
*Herminium monorchis*, SEM pre-anthetic developing flowers. (A–D) dissected gynostemia of different flowers, showing successive stages of bursicle development. (E, F) Same flower bud at different phases of dissection. (G–I) Lateral views of dissected flower buds, showing successive stages of spur development. Abbreviations: A1 = median stamen of outer whorl, au = auricle, bur = bursicle, lab = labellum, lc = lateral carpel, mcl = median carpel, ro = rostellum, sp = spur, st = stigmatic surface. Scales = 100 µm.

In *H. monorchis*, the lateral sepals are the first organs to become differentiated on the floral apex (Fig. 3A, 3B), separated by an adaxial region of tissue that will eventually become the labellum. At this stage, there is a common primordium for the median sepal and outer-whorl median stamen, which later becomes differentiated into two distinct primordia. The two lateral petals are initiated as bulges of tissue between the sepals. Spur development occurs later in ontogeny (Fig. 4E, 4G–4I), consistent with the developmental timing of spurs of other orchidoid genera (Bateman & Sexton, 2008; Box et al., 2008; Bell et al., 2009).

### Later development and anthesis (Figs. 5, 6)

**Figure 5:**
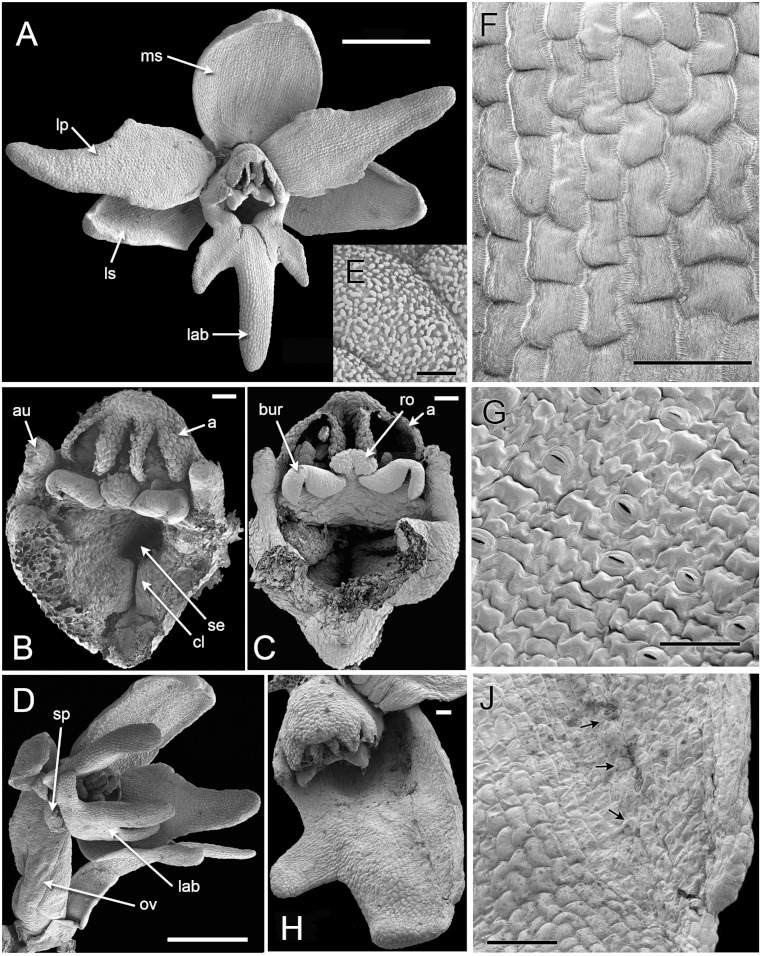
*Herminium monorchis*, SEM anthetic flowers. (A) Fully open flower. (B, C) Dissected gynostemia of open flowers. (D) Lateral view of open flower, showing spiral twisting of the ovary (resupination). (E) Surface of pollen massula, showing baculate-psilate sculpturing. (F) Surface of lower part of central lobe of open labellum, showing fine striations. (G) Surface of lateral sepal, showing stomata. (H) Teratological specimen in which one of the lateral sepals is fused with the labellum. (J) Detail of (H), showing boundary between fused labellum and sepal, with stomata (arrowed) on sepal region. Abbreviations: a = fertile anther, au = auricle, bur = bursicle, cl = carpel lobe, lab = labellum, lp = lateral petal, ls = lateral sepal, ms = median sepal, ov = ovary, ro = rostellum, se = spur entrance, sp = spur. Scales: A, D = 1 mm, B, C, F, G, J = 100 µm, E = 5 µm.

**Figure 6:**
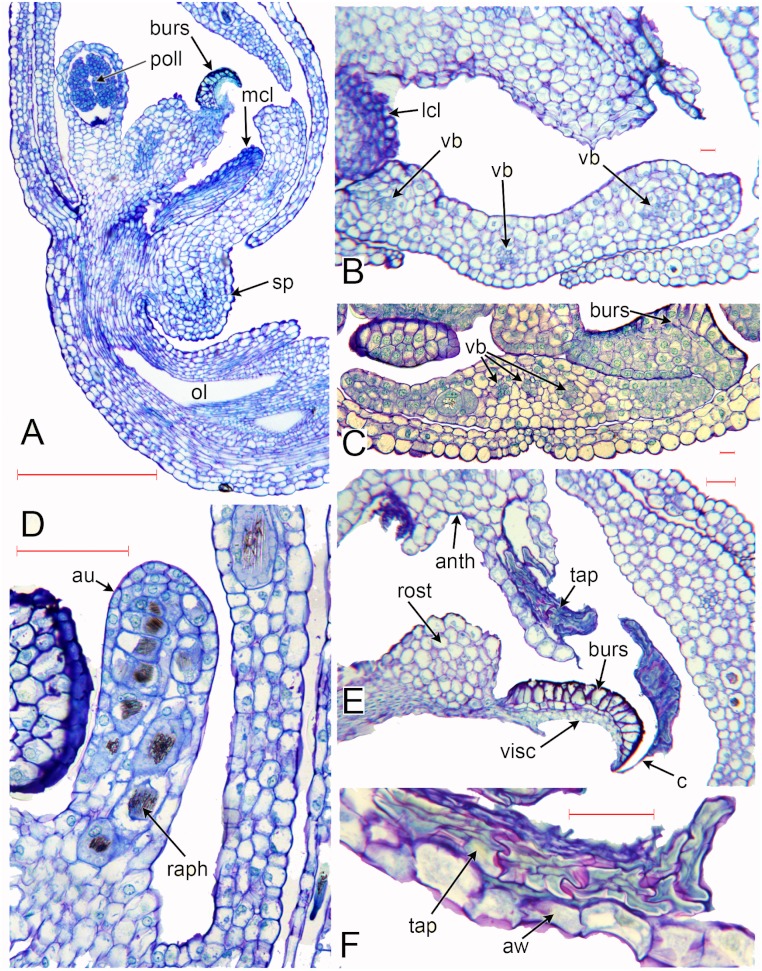
*Herminium monorchis*, light micrographs (LM) of flower sections. (A) Longitudinal section (LS) of flower, almost open. (B, C) Transverse sections (TS) of flower buds at similar stage to (A). (D) LS auricle, showing raphide crystals. (E) TS open flower, showing degenerating tapetum adherent to sticky viscidium, forming a caudicle attached to pollinium. (F) Detail of tapetum in (E). Abbreviations: anth = anther, au = auricle, aw = anther wall, burs = bursicle, c = caudicle, lcl = lateral carpel lobe, mcl = median carpel lobe (stigmatic surface), ol = ovary locule, raph = raphide crystals, sp = spur, tap = tapetum, vb = vascular bundle, visc = viscidium. Scales: A = 500 µm, B, C, E, F = 50 µm, D = 100 µm.

In *H. monorchis*, as in the majority of orchids, the inferior ovary has become spirally twisted by anthesis (Fig. 5D), making the flower resupinate. The six bright green perianth organs of the mature flower – three sepals and three petals – are similar in overall size (Fig. 5A). The sepals are more-or-less oval and each bears numerous stomata on the adaxial surface (Fig. 5G). The lateral petals are similar in size to the labellum, but they are much less deeply trilobed and entirely lack stomata. The labellum is narrow, markedly trilobed and bears a very short spur (Fig. 6A); the spur entrance lies between the two lateral carpel lobes (Fig. 5B, 5C).

The cells in the lower part of the middle lobe of the adaxial surface of the labellum are markedly striated (Fig. 5F). In common with the other petals, the labellum lacks stomata. We observed a teratological specimen in which the labellum lacked one of its lateral lobes and was fused with one of the lateral sepals; the two organs (labellum and lateral sepal) remained clearly distinguishable by their contrasting surface morphology, notably the presence or absence of stomata (Fig. 5H, 5J).

As in other orchidoids, *H. monorchis* possesses a single, fertile, erect anther of the outer-whorl median stamen (A1). Each of the two thecae contains a single pollinarium, which is a complex structure composed of the pollinium, viscidium and caudicle. The pollinium contains several massulae of baculate-psilate pollen. Each pollinium is linked to an adhesive disc (viscidium) via a short, thick mucilagenous thread, termed a caudicle (Fig. 6E, 6F). The caudicle is formed through the breakdown of the tapetum, whereas the sticky viscidium is formed from part of the bursicle. Two prominent auricles are located lateral to the fertile anther (Fig. 5B). Abundant idioblasts containing raphide crystals are distributed throughout the floral tissue, showing particularly strong concentration within the auricles (Fig. 6D).

Three distinct carpel apices are present; the median apex lies opposite the outer-whorl median stamen (A1) and is significantly larger than the two lateral carpel apices. The apex of the median carpel is itself distinctly trilobed. Its basal (proximal) part becomes increasingly papillate during development, and by anthesis it is partly recessed below the fertile anther. The middle lobe (apex) of the median carpel lobe forms the rostellum – an inverted U-shaped papillate bulge that expands into the gap between the two anther thecae, where they diverge slightly (Fig. 5B, 5C). The bursicles develop from the lateral projections of the median carpel lobe and expand into prominent, hook-shaped structures that largely enclose the paired triangular viscidia (Fig. 6A, 6C, 6E). The walls of the bursicles are thickened at anthesis (Fig. 6E).

### Vasculature (Figs. 6, 7)

**Figure 7:**
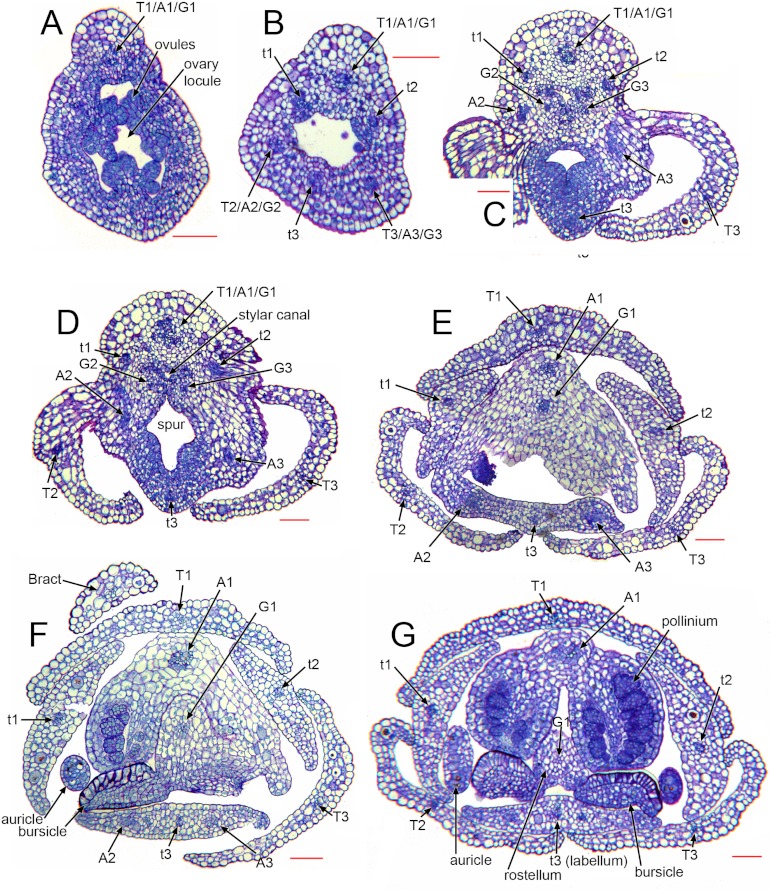
*Herminium monorchis*, light micrographs (LM) of flower sections, serial transverse sections (TS) through a single pre-anthetic flower bud, showing vasculature. Abbreviations: A = putative bundles of outer-whorl stamens (numbered as in Fig. 1), a = putative bundles of inner-whorl stamens (numbered as in Fig. 1), T = sepals, *t* = petals (*t*3 = labellum). Scales = 100 µm.

As in many other orchids (e.g. Swamy, 1948), the vasculature of the *Herminium* flower is difficult to trace precisely, especially in the condensed region toward the apex of the ovary, where all the organs are united (Fig. 7C–7E). Each of the six unbranched vascular traces that pass through the inferior ovary can be assigned to a corresponding perianth segment. The lateral petals and all three sepals each contain a single vascular trace, whereas the median petal (labellum) possesses three vascular traces (Figs. 6B, 6C, 7D–7G). The median trace of the labellum supplies the spur. The vascular traces that supply the median outer stamen (A1) and the median carpel diverge from the trace supplying the median sepal. The two lateral traces of the labellum each diverge from the traces supplying the two adjacent lateral sepals. Our study indicates that the two lateral labellum traces could represent the missing outer-whorl lateral stamen traces (A2, A3), because they diverge at the appropriate locations within the flowers.

## Discussion

### Structural homologies in orchid flowers: *Herminium* as a model

#### Bursicle hypothesis

Hagerup (1952) interpreted the prominent paired bursicles in *H. monorchis* as the “missing” staminodes A2 and A3, based on their large size, characteristic shape and his apparently mistaken report that they become fused with the rostellum and anther only at late stages of development. Indeed, our results show that the bursicle itself has (perhaps superficial) anther-like anatomy. The thick-walled epidermis of the bursicles resembles well-developed wall layers in fertile anthers, notably the endothecium, which contributes to both structural support and dehiscence. The wall thickenings in the bursicles could play a similar role to those in a fertile anther, breaking the thin membrane that surrounds the viscidia (Claessens & Kleynen, 2011). The thickenings may also assist removal of the bursicles; both the bursicles and viscidia of *Herminium* are sometimes entirely removed by pollinating insects (Hagerup, 1952; Claessens & Kleynen, 2011).

In *Herminium*, as in other Orchidoideae (Kurzweil, 1987b; Kurzweil, 1998), the apex of the median carpel is trilobed, and the bursicles develop from the lateral lobes, relatively late in flower ontogeny. In addition to their lack of vasculature and late development (both features that are rare, but not unknown, in stamen homologues), the location of the bursicles – situated on the sides of the median carpel lobe – argues against a homology between the bursicles and staminodes.

#### Auricle hypothesis

As in most other Orchidoideae, *H. monorchis* possesses a conspicuous pair of auricles. Several authors (e.g. Brown, 1833; Eichler, 1875; Hagerup, 1952) homologized these lateral sterile outgrowths of the gynostemium with the inner lateral stamens (a1, a2), based primarily on their location relative to other organs (reviewed by Kurzweil, 1998; Rudall & Bateman, 2002). In contrast, other observers (e.g. Swamy, 1948; Vermeulen, 1966) considered that the inner lateral stamens are “entirely absorbed” into the gynostemium in Orchidoideae. Kurzweil (1987b), Kurzweil (1998) and others interpreted the auricles of Orchidoideae as filament appendages rather than staminodes, based on their insertion on the fertile anther (the outer median anther, A1) rather than on the hypanthium. In contrast, Kurzweil (1988) and Kurzweil (1998) interpreted the hypanthial bulges that occur in some Epidendroideae as staminodial. In *H. monorchis*, the auricles are initiated earlier than the bursicles, but they too remain unvascularized. They contain strong accumulations of raphide crystals, similar to those reported by Kocyan & Endress (2001) in the staminodes of apostasioid orchids. Thus, both their anatomy and location opposite the lateral petals are consistent with – though do not conclusively demonstrate – a staminodial origin.

Teratological data are also highly conflicting. In support of the auricle hypothesis, Lindley (1853: 176) referred to “imperfect anthers occasionally appearing at the side of the perfect one” in orchidoid terata, suggesting that the auricles have been transformed into fertile stamens. On the other hand, there are many examples of teratological flowers of orchidoids in which several fertile stamens are present, each possessing supernumerary auricles (e.g. Brown, 1833; Vermeulen, 1953). In these cases, the additional stamens do not appear to represent transformed auricles.

#### Compound-labellum hypothesis

The compound-labellum hypothesis was first proposed by Brown (1833) and later endorsed by Lindley (1853) and Darwin (1862). Specifically, the “missing” outer lateral staminodes (A2, A3), and sometimes the inner median stamen (a3), were hypothesized to be integrated into the median petal. Charles Darwin considered that “this view of the nature of the labellum explains its large size, its frequent tripartite form and especially its manner of coherence to the column, unlike other petals” (Darwin, 1862: 294) . Primary support for this theory was derived from vasculature; each of the two lateral petals is supplied by a single vascular trace, but the labellum is supplied by three traces. No vestige remains of the inner median staminode (a3), but the two lateral labellum traces were historically thought to be the remnant vascular traces for the outer-whorl lateral staminodes (A2, A3). Our study indicates that in *H. monorchis*, the lateral labellum traces are derived from, and located in the same sector as, the lateral sepal traces, so they could conceivably represent the “missing” outer-whorl lateral staminode traces (A2, A3). However, there currently exists insufficient evidence to either endorse or refute this hypothesis.

With a few exceptions (notably Nelson, 1965; Nelson, 1967), most authors (e.g. Swamy, 1948; Vermeulen, 1966; Kurzweil, 1998) have rejected the compound-labellum hypothesis for orchids, basing their arguments on the existence of little supporting evidence and some apparently contradictory data (reviewed by Rudall & Bateman, 2002). In contrast, a compound labellum is widely accepted for members of another monocot order, Zingiberales, specifically composed of two united staminodes in Zingiberaceae and five united staminodes in Costaceae (Endress, 1994; Rudall & Bateman, 2004).

In his detailed study of the vasculature of orchid flowers, Swamy (1948) found that both labellum and stamen vasculature are often highly plastic, even within a single species, and compound stamen traces are sometimes present (e.g. a1 linked with A2, a2 linked with A3). In cypripedioid orchids, in which the two inner lateral stamens (a1, a2) are both fertile, the dorsal stamen bundles (a1, A1, a2) are all present, but the ventral stamen bundles (A2, a3, A3) are all absent, except in some teratological flowers. Swamy reported that only the A1 bundle is present in many monandrous orchidoids, including *Habenaria* s.l. – the genus within which *Herminium* is nested phylogenetically (Fig. 2C). In some other monandrous orchidoids, either the inner lateral stamen traces (a1, a2) or the outer lateral stamen traces (A2, A3) are sometimes present in the gynostemium, but rarely are both present.

In most orchids, the lateral labellum bundles are supplied by marginal veins from the adjacent lateral sepals, which form by dichotomies in the procambial strands. Similarly, in the putatively earliest diverging extant orchid lineage, Apostasioideae, which have poorly differentiated labella, the marginal traces of both the labellum and the two lateral petals are supplied by the marginal veins of the adjacent sepals (Kocyan & Endress, 2001; Rudall & Bateman, 2002). In a few orchidoid genera that produce two spurs per labellum, notably *Satyrium*, the spur vasculature is derived from the marginal labellum traces, which originate from the adjacent sepals (Swamy, 1948). Vascularization of petals from adjacent sepals (and *vice versa*) occurs frequently in flowers of both monocots and eudicots, a feature that is at least partly positively correlated with the breadth of the organ near its point of insertion (reviewed by Rudall & Bateman, 2002; Remizowa et al., 2012).

### Prospects for interpreting orchid flowers using evo-devo

Despite the increasing number of developmental genetic studies of orchids, knowledge of the nature and location of expression of key developmental genes remains sketchy. In contrast, the number of genes within each gene family present in orchids has become clearer, as a series of species spanning all but one of the five taxonomic subfamilies have yielded broadly similar results when analyzed. Orchids have proven to be typical of most angiosperms in yielding just one gene from the key floral transition gene *FLO/LFY* (e.g. Montieri, Gaudio & Aceto, 2004) and from the (mostly MADS-box) categories that permit A-, C-, D- and E-class functions of floral identity (e.g. Kanno et al., 2007; Tsai et al., 2008) – note that, in orchids, A-class genes apparently serve mainly to assist the vegetative-to-floral transition (Chen et al., 2007). In the rare cases where two such genes have been reported from a clade within the orchid family, they may be confined to a single species within that clade (A-class: Skipper et al., 2005). Most orchids also contain only a single copy of the *GLO/PI* subclass of B genes, the intriguing exception being subfamily Orchidoideae. Indeed, two *PI*-like copies have been found in two species of *Habenaria* (Kim et al., 2007; Pan et al., 2011), the polyphyletic genus within which *Herminium* is phylogenetically nested (Fig. 2C). In addition, over-expression of an orchid *GLO/PI*-subclass gene caused male sterility in tobacco (Guo et al., 2007).

The most notable general exception to this single-copy rule is B-class genes of the *DEF/AP3* subclass, which have consequently attracted disproportionate interest from orchid researchers (Tsai et al., 2004; Tsai et al., 2008; Kanno et al., 2007; Kim et al., 2007; Mondragón-Palomino & Theissen, 2009; Mondragón-Palomino & Theissen, 2011; Mondragón-Palomino et al., 2009; Chang et al., 2010; Pan et al., 2011). Three or, more often, four such genes have been found in each species, and there has been general agreement that differential expression of these genes is largely responsible for the characteristic differentiation of members of the two closely-spaced tripartite perianth whorls into three sepals of the outer whorl versus the inner whorl of two lateral petals and a median labellum – the most elaborate perianth member present in most orchid flowers (e.g. Rudall & Bateman, 2002).

There has been less agreement among developmental geneticists regarding the relative functions of the members of the four clades of *DEF*-like genes (cf. Mondragón-Palomino et al., 2009; Chang et al., 2010; Mondragón-Palomino & Theissen, 2011). In the elegant model advanced by Mondragón-Palomino et al. (2009) and later subtly modified by Mondragón-Palomino & Theissen (2011) and Pan et al. (2011), in all but the earliest stages of flower development, *DEF*-like clades 1 and 2 are expressed in both the outer and inner whorls, whereas clade 3 is confined to the inner whorl and clade 4 to the labellum (all B-class genes are expressed in the gynostemium, together with C- and D-class genes: Xu et al., 2006). The most parsimonious interpretation of these observations is that the four *DEF*-like clades are orthologous and reflect at least one whole-genome duplication, each duplication event being followed by neofunctionalization of at least one copy. Remarkably, all copies were retained in the lineage and underwent transcriptional divergence, thus conferring “modularization” and independent evolutionary fates on the three categories of perianth segment – this occurring despite the strong overall canalization of the archetypal orchid flower.

Teratological flowers occur in relatively large numbers in orchids (e.g. Rudall & Bateman, 2003; Bateman & Rudall, 2006; Duttke, Zoulias & Kim, 2012). For example, in this study, we observed a flower in which the labellum was congenitally fused with one of the lateral sepals. The fact that the sepals in *Herminium* are unusual among orchid flowers in developing stomata (and, being green, are probably photosynthetic) allowed us to clearly distinguish the identities of the respective organs. When combined with gene expression studies, investigations of teratological flowers help to improve our understanding of the orchid flower. For example, Duttke, Zoulias & Kim (2012) reported an extraordinary mutant collection in the Wind Orchid (*Neofinetia falcata*: Epidendroideae), though these mutations were described primarily for the perianth rather than for the fertile structures. Pan et al. (2011: 1516) fundamentally misunderstood the categorization of orchid floral mutants that was initiated by Bateman (1985), detailed by Bateman & Rudall (2006) and further amended by Mondragón-Palomino & Theissen (2011). Nonetheless, their multiple comparisons of pairs of wild-type and mutant floral morphs distributed across much of the orchid family allowed Pan et al. (2011: 1527) to reach the important conclusion that “the major critical transition points represented by the asynchrony of relocated expression in duplicated *AP3* paralogs implies their dualistic roles in floral organ specification and indicates that the shifting patterns of *AP3* genes may determine the fate of orchid perianth growth and development, both temporally and spatially.” We suspect that heterochronic shifts in the precise timing of expression of *DEF/AP3* genes are capable of generating heterotopic phenotypes, at least one member of one category of floral organ replacing another.

We reluctantly conclude that gene expression studies in orchid flowers may prove unable to fully untangle the precise organ homologies of the gynostemium and the perennial mystery of the “missing” stamens, because the structures concerned are so profoundly congenitally united throughout flower development.

### Profound integration of orchid floral organs

As this study and others have clearly shown, there is no simple answer to determining the homology of the orchid labellum. We consider it highly unlikely that the bursicles are directly staminodial, but equally we find no strong evidence that either the auricles or labellum incorporate staminodia. The existence of much contradictory data for each of the hypotheses examined in this paper raises the question of whether the profound integration of orchid floral organs could have resulted in a high degree of plasticity, even in different lineages within orchids. Crucially, the exceptional loss of organ boundaries displayed by orchid flowers could be associated with considerable overlap in gene expression.

Organs can be united either postgenitally or congenitally, and both phenomena are especially frequent in flowers (Endress, 1994). Postgenital fusion of developing organs that become closely appressed after initiation is a developmental process that was termed “surface fusion” by Sattler (1978) (see also Verbeke, 1992). In *Arabidopsis*, postgenital organ fusion results from contact-mediated cell adhesion and possible reprogramming of epidermal cell fate, in a localized response involving genes such as *FIDDLEHEAD* or *HOTHEAD* (Lolle & Pruitt, 1999; Pruitt et al., 2000; Krolikowski et al., 2003). In contrast, some organs are integrally united from inception and hence the organ boundaries are never fully specified – a developmental pattern that is more aptly termed “congenital union” (Verbeke, 1992) or “integration” than the more widely used term “congenital fusion.” Sattler (1978) and Verbeke (1992) identified different types of congenital union (including zonal growth), and more recent studies suggest the involvement of several genes (e.g. Lee, Geisler & Springer, 2009).

Both postgenital fusion and congenital union of carpels are widespread among monocots, many species showing a combination of both processes (Rudall, 2002; Remizowa, Sokoloff & Rudall, 2010). In orchids, postgenital fusion occurs between the lateral sepals of cypripedioids to form a complex “synsepalum” (Kurzweil, 1993), and the anthers of *Apostasia* fuse postgenitally into a tube around the style (Kocyan & Endress, 2001). Conversely, a high degree of congenital union is present in the gynostemium of the more derived groups of orchids possessing well-developed gynostemia, notably the species-rich epidendroid and orchidoid groups, which include *H. monorchis*. This trend is well illustrated by those epidendroid orchids that produce what have been termed cuniculoid nectar spurs, employing the lower surface of the gymnostemium as the roof of the spur (cf. Dressler, 1993: 30). In the derived orchid groups, throughout flower development there exists profound integration between all the floral whorls, from the base of the gynostemium to the base of the ovary. Such comprehensive union between organs of different whorls is rare in angiosperms, and (with a few exceptions) most examples are congenital, resulting from zonal growth (Verbeke, 1992). A similar – perhaps related – example of highly cryptic organ integration occurs in some pseudomonomerous gynoecia, in which some of the fused carpels are sterile (reviewed by González & Rudall, 2010). In extreme cases of pseudomonomery, such as the grasses (Philipson, 1985; Rudall et al., 2005), a gynoecium that is putatively derived from a congenitally united multicarpellate ovary appears unicarpellate but retains some supposedly atavistic features.

On the other hand, orchid flowers display relatively pronounced bilateral symmetry. Unfortunately, there have not yet been detailed studies of TCP genes in orchids, which have been shown to strongly influence bilateral symmetry in eudicot families (e.g. Cubas, 2002) and possibly also in grasses (Yuan et al., 2009), and could therefore be implicated in generating the extreme bilateral symmetry that characterizes every whorl of almost all orchid flowers (Rudall & Bateman, 2002).

### Partial homology and *KNOX* gene expression: a potential new model for orchid flower development

There exists some ambiguity in the terms “compound” and “simple” when applied to plant organs. In general, a floral structure is considered to be compound if it is formed from more than one organ. For example, a syncarpous ovary is a compound structure formed from three carpels that are either postgenitally fused or congenitally united (sometimes cryptically so, as in grasses). Conversely, a compound leaf bearing several leaflets is treated as a single organ, despite its relative morphological complexity compared with a – typically unlobed – simple leaf; a deeply lobed leaf is arguably intermediate between these two conditions. The apparent difficulty in unequivocally determining whether a structure is simple or compound results from the application of a strictly typological approach to organ homologies. This conundrum was addressed by Sattler’s concepts of “partial homology” and “continuum morphology” or “process morphology” (e.g. Sattler, 1990; Sattler, 1992; Sattler, 1994; Sattler & Jeune, 1992, see also Fisher & Rutishauser, 1990), which emphasize the dynamic aspect of plant form – the compound leaf is considered intermediate between a simple leaf and a leafy shoot. Interestingly, there is some genetic basis for these concepts; for example, *KNOX* genes not only play a role in shoot meristem maintenance and organization but are also implicated in compound leaf development (e.g. Bharathan & Sinha, 2001; Hay & Tsiantis, 2009; Hay & Tsiantis, 2010). *KNOX* genes can induce lobed and compound leaf phenotypes when they are constitutively expressed in simple-leaved species.

Orchid flowers possess structures that are compound in all senses of the term. As we have already discussed, the profound integration of multiple orchid floral organs into a gynostemium makes untangling their homologies especially problematic. We suggest that the orchid labellum can credibly be analogized with a compound leaf (i.e. a compound phyllome), because it is typically a fundamentally lobed structure. Moreover, there is now some evidence of *KNOX* function in labellum development in the orchidoid species *Dactylorhiza fuchsii* (Box et al., 2012), in which the labellum is three-lobed and bears a prominent spur (Box et al., 2008; Bell et al., 2009). This remarkable new genetic finding supports earlier preliminary evidence of a role for *KNOX* genes in spur development in other angiosperms (Golz, Keck & Hudson, 2002; Box et al., 2011). This indirect evidence indicates that *KNOX* genes could be implicated not only in spur development but also in the development of the characteristic, and often elaborate, lobed morphology of the orchid labellum.

Even more intriguing is the possibility of a role for *KNOX* genes in determining the morphology of non-labellar organs in the orchid flower, in addition to the labellum. In orchid groups such as Disinae that generate spurs from sepals rather than petals, the spur is present on organs that lie in (or, at least, overlap with) the same floral sector as the labellum but occupy a different (slightly earlier-formed) floral whorl. This apparent longitudinal displacement is presumably caused by differential timing of gene expression – in other words, a heterotopic pattern reflecting a heterochronic process. Our data suggest that (as in many other orchids) differentiation of the rostellum and bursicles in *Herminium* results from deep trilobing of the apex of the median carpel, which is highly reminiscent of the trilobing of the labellum. Such carpel lobing also suggests differential – perhaps prolonged – timing of gene expression during floral ontogeny. A role for *KNOX* genes remains highly speculative. However, if confirmed, timing of *KNOX* expression could be crucial in establishing the diverse range of floral morphologies that at least partly accounts for the exceptional species richness exhibited by orchids. The much-researched functional morphology of the orchid flower could therefore reflect extreme synorganization and the associated overlap in gene expression between organs.
